# Knockdown of lncRNA HNF1A-AS1 inhibits oncogenic phenotypes in colorectal carcinoma

**DOI:** 10.3892/mmr.2019.10373

**Published:** 2019-06-11

**Authors:** Wenyi Zhu, Peipei Zhuang, Wen Song, Shiyu Duan, Qiong Xu, Man Peng, Jun Zhou

Mol Med Rep 16: 4694-4700, 2017; DOI: 10.3892/mmr.2017.7175

Following the publication of the article, the authors noted an error associated with the presentation of Fig. 3B. This Figure part showed the cell cycle analysis as determined by flow cytometry of HT29 cells transfected with either a HNF1A-AS1-specific siRNA or a negative control siRNA at 48 h post-transfection. An error was made in the compilation of this Figure, and the same data were inadvertently selected to represent the cell cycle analysis of both the HT29-SI and the HT29-NC cells (i.e., the data relating to the HT29-NC cells were included in the Figure twice). A corrected version of Fig. 3 is shown on the next page. Note that this correction affects neither the interpretation of the data nor the reported conclusions of this work.

The authors all agree to this Corrigendum, and regret that this error went unnoticed during the proofs correction stage. They also wish to thank the Editor for allowing them the opportunity to publish this Corrigendum, and regret any inconvenience this error has caused.

## Figures and Tables

**Figure 3.**
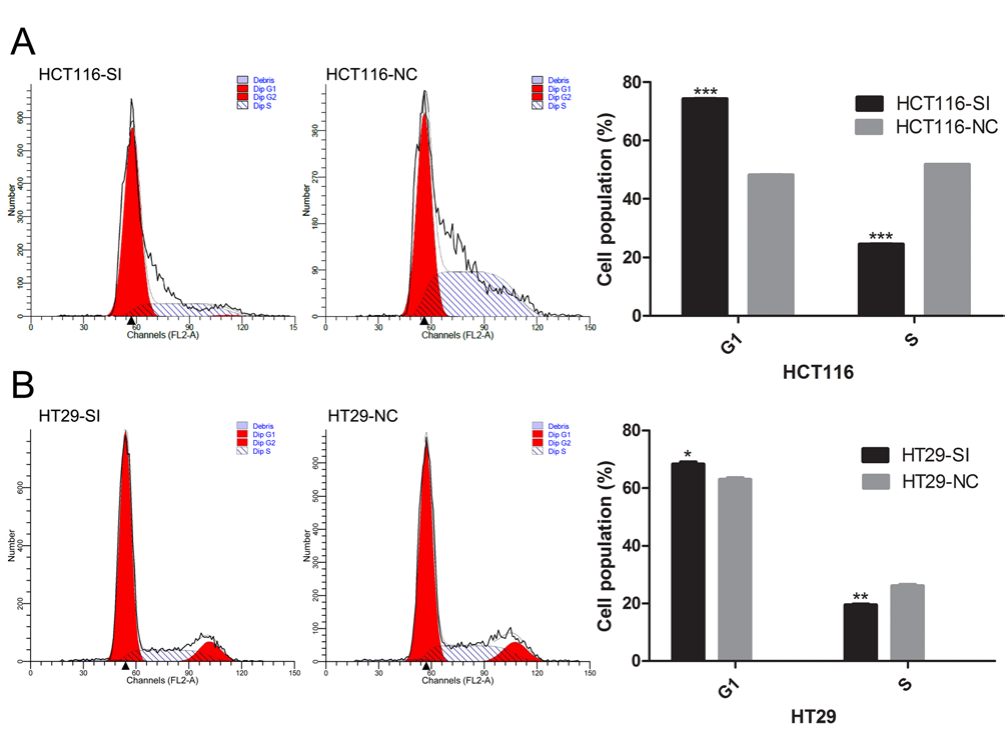
Effect of HNF1A-AS1 knockdown on CRC cell cycle progression *in vitro*. (A) HCT116 and (B) HT29 cells were transfected with either a HNF1A-AS1-specific siRNA or a negative control siRNA, and cell cycle analysis was performed at 48 h post-transfection by flow cytometry. Data are presented as the mean ± standard deviation from three independent experiments. *P<0.05, **P<0.01 and ***P<0.001 vs. NC. HNF1A-AS1, HNF1A antisense RNA 1; CRC, colorectal carcinoma; siRNA, small interfering RNA; SI, siRNA; NC, negative control.

